# Hydration kinetics of direct expanded *tef* flour breakfast cereals in water and milk

**DOI:** 10.1002/fsn3.80

**Published:** 2013-12-08

**Authors:** W K Solomon

**Affiliations:** 1 Department of Food Technology and Process Engineering, Haramaya UniversityHaramaya, Ethiopia; 2 Department of Consumer Sciences, University of SwazilandLuyengo, Swaziland

**Keywords:** Breakfast cereals, *Eragrostis tef*, extrusion, hydration

## Abstract

Hydration kinetics of *tef* flour breakfast cereals extruded at barrel temperatures of 110, 130, and 150°C was investigated by hydrating them in water and whole milk at 25°C (±1°C). The normalized Weibull model described the rehydration characteristics of the extrudates in water and milk adequately (*R*^2^ = 0.98–0.99). Water absorption rate was significantly (*P* < 0.05) influenced by barrel temperature where extrudates processed at 150°C barrel temperature exhibited high water absorption rate followed by those extruded at 130 and 100°C, respectively. Hydration rate and equilibrium moisture content were higher for samples hydrated in water than those in milk. In view of the values of the shape parameter *β*, the hydration process is predominantly controlled by diffusion (*β* = 0.40–0.51) for samples extruded at 110°C whereas external resistance to mass transfer dominated (*β* = 0.60–0.73) those extruded using 150 and 130°C. Extrudates processed at 130 and 150°C exhibited better hydration characteristics. Thus, these temperature ranges could be used to produce extruded products from *tef*.

## Introduction

*Tef* (*Eragrostis tef*) is an important cereal crop indigenous to Ethiopia comprising about 20% of cereal production providing the major daily calorie for the vast majority of the population (Umeta and Faulks 1988; Ketema 1997; Bultosa and Taylor 2004). *Tef* seed is a millet-like, tiny, and prolate spheroid with average length and width of 1.01 and 0.59 mm, respectively (Zewdu and Solomon 2007). The proximate composition (% d.b.) of *tef* seed is reported to be 9.4–13.3 protein, 73 carbohydrate, 1.98–3.5 crude fiber, 2.0–3.1 fat, and 2.7–3.0 ash (Bultosa and Taylor 2004). Despite its major contribution, the processing techniques and the types of food products made from *tef* remain traditional. Recently, however, there is growing interest to develop new products from *tef* using modern processing techniques like extrusion cooking to harness its potential.

Extrusion-cooked direct expanded breakfast cereals have cellular-like structures mainly formed by air pockets surrounded by walls of gelatinized starch that contribute to texture and hydration capacity (Bhattacharya 1997; Machado et al. 1998). Breakfast cereals are commonly consumed by soaking in milk (Sacchetti et al. 2003, 2005) where the product readily takes up moisture resulting in undesirable changes, especially in the most desirable and primary quality attributes like crispiness, brittleness, and crunchiness due to the plasticization effect of water (Fontanet et al. 1997; Machado et al. 1999; Sacchetti et al. 2003; Lewicki 2004; Gondek and Lewicki 2006). In view of this, the hydration characteristic of breakfast cereals and other dried food products is recognized as an important quality attribute and recently has received increasing interest (Machado et al. 1997, 1998, 1999; Sacchetti et al. 2003, 2005; Lucas et al. 2007).

Previous studies on the quality of extruded breakfast cereals and snacks focused mainly on the effect of operating conditions on some physicochemical and functional properties and sensory qualities. High extrusion temperature has been reported to result in less compact, porous, and better expanded products (Anderson and Hedlund 1990; Ilo et al. 1996; Lo et al. 1998; Sacchetti et al. 2004; De Pilli et al. 2005), which have better hydration properties. Water absorption index (WAI) and water solubility index (WSI) are regarded as measures of hydration capacity (power), and high extrusion temperatures were found to generally increase WAI and WSI (Kirby et al. 1988; Ollett et al. 1990; Sacchetti et al. 2004; Ding et al. 2006), thereby increasing the water absorption. The degree of gelatinization of starch, which is also linked to the water absorption capacity (Gambus et al. 1999; Cheyne et al. 2005), increased with increase in extrusion temperature (Kirby et al. 1988; Ilo et al. 1996; Ding et al. 2005).

Describing and modeling the kinetics of moisture uptake by breakfast cereals could lead to a better understanding and control over the mechanisms underlying the sorption process and the ability of the product to rehydrate that could help in process optimization (Machado et al. 1997, 1998, 1999; Sacchetti et al. 2003; Lucas et al. 2007). Nonetheless, limited studies have been carried out in this regard. Previous hydration kinetic studies on breakfast cereals indicated that water absorption rate increases with increase in hydrating media temperature (Machado et al. 1998, 1999) and decreases with increase in fat and total solid content of milk used as hydrating medium, and amount of coating sugar (Machado et al. 1997, 1999; Sacchetti et al. 2003). These studies indicated that the rate of moisture uptake depends on the physicochemical properties and structural characteristics of products, which in turn are influenced by the extrusion processing conditions. In view of few studies in the past, limited information is available in the literature on the influence of extrusion operating conditions on the kinetics of moisture uptake by breakfast cereals. Sacchetti et al. (2005) reported that the hydration rate of cereal-based breakfast cereals increased with increase in extrusion temperature from 100 to 110°C. However, studies over a wider span of processing conditions covering the ranges in which breakfast cereals are produced are required. Particularly, there is limited information on extruded products made from *tef* flour. The effect of barrel temperature (110, 130, and 150°C) on the rehydration kinetics of *tef* flour extrudates in water for DZ-01-196 cultivar was investigated (Solomon 2008). However, different cultivars due to their proximate composition behave differently during hydration. Moreover, breakfast cereals are commonly consumed after hydrating in milk. Thus, hydration studies in milk are important. The objectives of this study were to investigate the effect of hydration medium and barrel temperature on the hydration characteristics and to model hydration kinetics of breakfast cereal made from *tef* flour (DZ-01-99 cultivar) in water and milk.

## Material and Methods

### Sample preparation

Flour from *tef* grain (variety DZ-01-99) grown at the Debre Zeit Agricultural Research Center, Ethiopia, during the 2005–2006 cropping season was used. The proximate composition of the flour used in this study (g/100 g) was found to be 10.69, 2.50, and 2.52 for crude protein, crude fat, and ash, respectively (Kebede 2006).

### Extrusion process

Extrusion was carried out using a pilot scale co-rotating twin screw extruder (model Clextral, BC-21 No. 124, Firminy, France) having 25 mm screw diameter. The barrel has a smooth 300 mm useful length and consists of three 100-mm-long modules. The temperature of the modules is regulated by electrical heating and water cooling system, and the temperature at each zone was controlled by a Eurotherm controller (Eurotherm Ltd., Worthing, UK). The flour was fed to the extruder inlet using a twin screw volumetric feeder (type KMV-KT20) and water at ambient temperature was injected into the extruder via an inlet port by a positive displacement pump (DKM-Clextral). The extruder die has four circular openings where the diameter tapers from 5 to 2 mm in a length of 9 mm. Extrusion was carried out at 110, 130, and 150°C barrel temperature, 17% (w.b.) feed moisture content, 9 kg/h feed rate, and 140 rpm screw speed. The operating conditions were selected based on a preliminary study and recommended values in the literature (Bouvier 2001). The extruded samples, cut to specific length, were dried in a hot air cabinet dryer at 150°C for 10 min to improve the crispiness (Bouvier 2001; Kebede 2006). The initial moisture content of the extrudates was 3.8% (d.b.).

### Hydration tests

About 2 g samples were kept in empty beakers and conditioned to the rehydration temperature of 25°C in a hot air oven before each test (Hung et al. 1993; Maskan 2002). After conditioning, 100 mL distilled water or whole milk at 25°C was poured into the beakers and then placed in a constant temperature water bath (Model 25; Precision Scientific Inc., Chicago, IL) set at 25°C (±1°C). The rehydration temperature falls in the ranges used in earlier moisture uptake studies of breakfast cereals (Machado et al. 1997, 1998, 1999; Sacchetti et al. 2003; Lucas et al. 2007). The chemical composition of milk used was determined and was found to be 2.73, 3.92, 13.79 g/100 g for fat, protein, and total solids, respectively. At time intervals ranging between 5 and 120 min (depending on soaking duration), the beakers were removed from the water bath and drained, and the samples were weighed using an analytical balance (CP 124S, Data Weighing Systems, Inc., Gottingen, Germany) with 0.1 mg accuracy. The samples were then dried in a hot air oven at 130°C for 4 h based on preliminary studies and in reference to Gondek and Lewicki (2006) to determine the moisture content. Each test was repeated four times to determine the mean values.

### Data analysis

The probabilistic normalized Weibull model (eq. 1) has been found to adequately describe the hydration characteristics of a variety of dry food products including breakfast cereals (Ilincanu et al. 1995; Cunha et al. 1998; Machado et al. 1998, 1999; Sacchetti et al. 2003, 2005; Marabi and Saguy 2004).


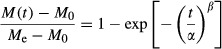
(1)

where *M*(*t*) is moisture content at time *t*, *M*_e_ is equilibrium moisture content after prolonged soaking, *M*_0_ is initial moisture content, *α* is the scale (rate) parameter, *β* is the shape parameter, and *t* is soaking time. The scale (rate) parameter represents the time needed to accomplish approximately 63% of the moisture absorption process whereas the shape parameter is a behavior index which is linked to the mechanism controlling the water absorption process (Machado et al. 1997; Cunha et al. 1998).

The parameters *β*, *α*, and *M*_e_ in the model were determined by nonlinear regression using JMPIN 5.0.1 statistics software (SAS 2002, JMP Version 5; SAS Institute Inc., Cary, NC). The degree of fit of the model was evaluated based on the values of coefficient of determination (*R*^2^) and root mean square error (RMSE) (eq. 2) and by examining the distribution of the residuals.


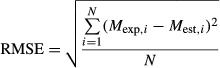
(2)

where *M*_exp_ and *M*_est_ are experimental and estimated moisture content, respectively, and *N* is the number of data points.

## Results and Discussion

### Hydration phenomena

**Figure 1:**
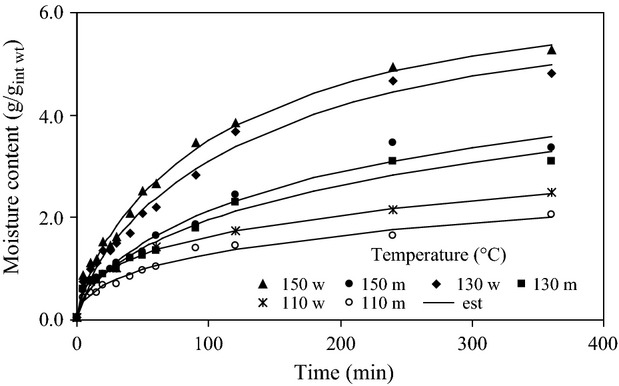
Experimental and estimated rehydration curves in water and whole milk at different barrel temperatures (m, milk; w, water).

The rehydration data of extrudates processed using different barrel temperatures and hydrated in water and whole milk are presented in Figure 1. Water absorption occurred at a higher rate in the early stages (60–90 min) of the rehydration process followed by decreasing rate and finally tending to cease at prolonged soaking time indicating the rehydration approached equilibrium condition. This trend was similar for samples extruded using 130 and 150°C barrel temperatures. For samples extruded using barrel temperature of 110°C water absorption continued steadily till the end of the hydration time and approaching equilibrium required more time. Water absorption rate depends on the difference between the water content at a given time and at saturation, which is the driving force. As hydration proceeds, the water content of the extrudates increased, thereby decreasing the driving force and consequently the water absorption rate. The process ceases when the extrudates attain the equilibrium moisture content. Similar characteristic water absorption trends have been reported for ready-to-eat and puffed breakfast cereals immersed in water or milk (Machado et al. 1997, 1998, 1999; Sacchetti et al. 2003, 2005; Lucas et al. 2007; Solomon 2008).

Samples extruded using barrel temperature of 150°C exhibited higher water absorption rate followed by those extruded at 130 and 110°C, which is apparent from the slope of the fitted lines particularly in the early stages (about the first 60–90 min) of the rehydration process (Fig. 1). This trend was similar while hydrating samples both in milk and in water. The trend observed in this study is in agreement with Solomon (2008) where *tef* extrudates processed at 150°C exhibited higher hydration rate than those extruded at 130 and 110°C. Sacchetti et al. (2005) also reported that extrudates processed at 110°C extrusion temperature exhibited higher hydration rate than those extruded at 100°C. Gelatinization, which is the conversion of starch to a cooked and digestible material by the application of heat and water, is one of the important effects extrusion has on the starch component of foods (Ilo et al. 1996; Cheyne et al. 2005; Ding et al. 2005, 2006). Water is absorbed and bound to the starch molecules inducing change in the starch molecule structure. The fact is that high extrusion temperature increases the degree of starch gelatinization (Ilo et al. 1996; Bhattacharya 1997; Ding et al. 2005; Singh et al. 2007), and water absorption rate and water absorption capacity by gelatinized starch are higher than those for raw starch (Gambus et al. 1999; Cheyne et al. 2005), explaining the high water absorption rate exhibited by extrudates processed at barrel temperature of 150°C followed by those extruded at 130 and 110°C, respectively. Earlier studies also revealed that high extrusion temperature reduces melt viscosity (Fletcher et al. 1985; Ilo et al. 1996; Lo et al. 1998; Ding et al. 2006), resulting in more expanded, less compact, and more porous breakfast cereals and snacks having air cells due to release of superheated steam (Fletcher et al. 1985; Anderson and Hedlund 1990; Ilo et al. 1996, 1999; Bhattacharya 1997; Guha et al. 1997; Ding et al. 2005; De Pilli et al. 2005), leading to less resistance to mass transfer and hence increased rate of water absorption.

It is also worth noting that water absorption rate in water is significantly higher than that in milk. This is evident from the trends and slopes of the fitted lines (Fig. 1), which were found to be significantly higher in water than those in milk. The lower water absorption rate of extrudates hydrated in milk could be attributed to the solids in milk, which block the pores of the extrudates, thereby reducing water absorption rate. Moreover, the fat component in the milk could also influence water absorption by accumulating on the surface of the extrudates (Machado et al. 1999). Hindrance to water absorption could be aggravated further due to the hydrophobic nature of fat adhered to the extrudates (Machado et al. 1998).

### Modeling rehydration kinetics

**Figure 2:**
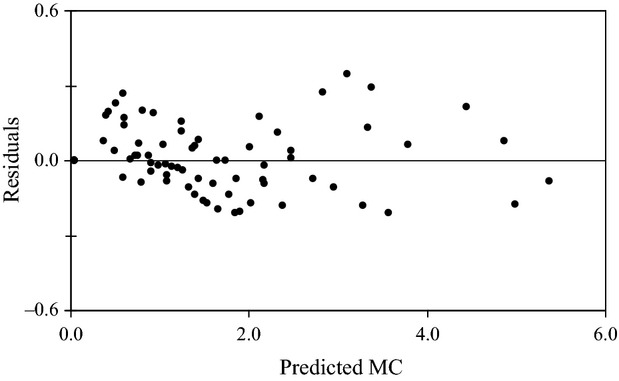
Plot of predicted moisture contents versus residuals.

The results of nonlinear regression including the scale (rate) parameter, *α*, the shape parameter, *β*, and equilibrium moisture content, *M*_e_ in equation 1, and the degree of fit in terms of *R*^2^ and RMSE are presented in Table 1. The results revealed that the normalized Weibull model adequately described the rehydration characteristics of *tef* flour breakfast cereals in water and milk with high *R*^2^ (0.98–0.99) and small RMSE values. Besides the quantified values of *R*^2^ and RMSE, the adequacy of the model was evaluated by the residual plot (Fig. 2). The residuals were found to be randomly distributed, demonstrating that the model adequately described the rehydration kinetics of the extrudates. Earlier studies have also shown that the normalized Weibull model adequately described the rehydration kinetics of puffed breakfast cereals and other dried products (Ilincanu et al. 1995; Cunha et al. 1998; Machado et al. 1998, 1999; Sacchetti et al. 2003, 2005; Marabi and Saguy 2004; Solomon 2008).

**Table 1:** Parameters and degree of fit in normalized Weibull model at different barrel temperatures.

Temperature (°C)	In water					In milk				
	*α* (min)	*β*	*M*_e_	*R*^2^	RMSE	*α* (min)	*β*	*M*_e_	*R*^2^	RMSE
110	2309.7	0.40	6.48	0.994	0.0424	381.65	0.51	3.22	0.981	0.084
130	150.59	0.73	5.88	0.986	0.1863	335.63	0.60	5.05	0.979	0.1605
150	123.47	0.73	6.05	0.992	0.1497	229.22	0.66	4.81	0.976	0.1684

*M*_e_, equilibrium moisture content; *α*, scale (rate) parameter; *β*, shape parameter; RMSE, root mean square error.

The value of *α*, which represents the time required to achieve 63% of the maximum moisture uptake (1 − e^−1^), was determined to be 2309.7, 150.59, and 123.47 min for samples extruded at 110, 130, and 150°C, respectively, indicating that the samples hydrated in water and extruded at barrel temperature of 150°C had a high water absorption rate followed by those extruded using 130 and 110°C barrel temperature. The trend was similar for extrudates hydrated in milk (Table 1). Similar trends of the effect of extrusion temperature on the hydration rate of breakfast cereals have been reported (Sacchetti et al. 2005). The value of *α* was found to be higher in milk than that in water (Table 1), demonstrating that the water absorption rate of samples hydrated in milk is less than that in water. This is also evident from the trends of the fitted lines (Fig. 1). Higher values of *α* indicate low water absorption rate or more time is required to reach 63% of the equilibrium moisture content (Ilincanu et al. 1995; Machado et al. 1998). The solids and fat in the milk coupled with less amount of free water could hinder the mass transfer process (Machado et al. 1997, 1999), resulting in lower degree of hydration of the extrudates hydrated in milk than in water. It was also visually observed that extrudates hydrated in water swell more and their structure was lost relatively easily, thereby decreasing the resistance to mass transfer whereas those hydrated in milk had a better retention of structure thus decreasing the water absorption rate. A similar phenomenon was reported for puffed breakfast cereals hydrated in water and milk (Machado et al. 1997). The water absorption rate of *tef* appeared to be less as compared to commercial puffed corn breakfast cereals (*α* = 32 min) (Machado et al. 1997, 1999) hydrated in water at 25°C, cereal-based breakfast cereals (*α* = 32.5 to 6.7 min) (Sacchetti et al. 2005), and corn flakes and flakes made from a mixture of cereals (*α* = 15.97 and 60.28 min) (Sacchetti et al. 2003) hydrated in semiskimmed milk. This difference could be attributed to the high bulk density of *tef* extrudates, which was 1380, 610 and 490 kg/m^3^ at barrel temperature of 110, 130, and 150°C, respectively (Kebede 2006), compared with most corn and rice-based commercial breakfast cereals where the bulk density ranges from 90 to 320 kg/m^3^ (Ilo et al. 1996, 1999; Guha et al. 1997). However, the hydration rate of *tef* extrudates processed at 130 and 150°C was higher than that of commercial peanut butter breakfast cereals (*α *= 503 min) hydrated in water at 25°C (Machado et al. 1998), which could be attributed to the fat content of the peanut butter breakfast cereals.

The value of the shape parameter, *β*, ranged between 0.40 and 0.73 (Table 1), indicating that the process is predominantly controlled by diffusion for extrudates processed at 110°C, whereas external resistance to mass transfer dominated for those extruded at 130 and 150°C (Cunha et al. 1998). Higher values of *β* (preferably more than 1) correspond to the lag time for moisture uptake (Machado et al. 1998) and are desirable with regard to the maintenance of crispness during rehydration (Machado et al. 1997). Thus, extrudates processed at 130 and 150°C barrel temperature would maintain better crispness during rehydration.

The equilibrium moisture content, *M*_e_, generally increased with an increase in barrel temperature (Table 1), which is also apparent from the trends observed in Figure 1. This phenomenon could be attributed to less expanded, less porous, and dense internal structure of the extrudates extruded at low temperatures (Fletcher et al. 1985; Anderson and Hedlund 1990; Ilo et al. 1996, 1999; Bhattacharya 1997; Guha et al. 1997; De Pilli et al. 2005) that reduces the water-holding capacity. However, extrudates processed at 110°C barrel temperature and hydrated in water appeared to have high moisture content (Table 1). Despite its statistical reliability, this high *M*_e_ value should be considered cautiously. In view of the steadily continued water absorption observed during the experimentation by samples extruded at 110°C and the associated *α* value (2309.7 min), it was not possible to approach equilibrium condition. Therefore, it is possible to arrive at such a high estimated equilibrium moisture content value. When the experimental data depart from equilibrium, the precision of the parameter's estimate decreases and *M*_e_ tends to be overestimated (Cunha et al. 1998; Machado et al. 1998).

The equilibrium moisture content of extrudates hydrated in whole milk was found to be slightly lower than those hydrated in water (Table 1). This could be attributed to the role of the fat and solids, which might have blocked the pores, resulting in smaller hydration or water-holding capacity. In earlier studies on rehydration of puffed breakfast cereals, the equilibrium moisture content of samples hydrated in water was twice the value of those hydrated in milk (Machado et al. 1997). Similarly, the increase in the concentration of total solids and fat content of milk has been reported to decrease the equilibrium moisture content of ready-to-eat breakfast cereals (Machado et al. 1999), which further explains the influence of solids and fat content on the hydration (water-holding) capacity.

## Conclusions

The water absorption rate and water absorption capacity (equilibrium moisture content) increased with an increase in barrel temperature from 110 to 150°C. The normalized Weibull model adequately described the water absorption characteristic of *tef* flour breakfast cereals hydrated in water and milk. Hence, the model could be used for prediction and process optimization purposes, though it lacks theoretical basis. Water absorption rate and equilibrium moisture content were higher for extrudates hydrated in water than those hydrated in milk. In view of the values of the shape parameter *β* (0.40–0.73), the rehydration process is predominantly controlled by diffusion for samples extruded at 110°C, whereas external resistance to mass transfer dominated for those extruded using 150 and 130°C. Extrudates processed at 130 and 150°C barrel temperature could maintain better crispness during rehydration. Hence, this temperature range might be preferred for producing *tef* flour breakfast cereals.
